# Abnormal protein post-translational modifications induces aggregation and abnormal deposition of protein, mediating neurodegenerative diseases

**DOI:** 10.1186/s13578-023-01189-y

**Published:** 2024-02-12

**Authors:** Wei Li, Hong-Lian Li, Jian-Zhi Wang, Rong Liu, Xiaochuan Wang

**Affiliations:** 1 Department of Pathophysiology, School of Basic Medicine, Key Laboratory of Education Ministry of China for Neurological Disorders, Tongji Medical College, Huazhong University of Science and Technology, Wuhan, 430030 China; 2 Co-innovation Center of Neuroregeneration, Nantong University, Nantong, 226001 JS China; 3 Shenzhen Huazhong University of Science and Technology Research Institute, Wuhan, China

**Keywords:** Protein post-translational modifications (PPTMs), Neurodegenerative diseases, Treatment strategy

## Abstract

Protein post-translational modifications (PPTMs) refer to a series of chemical modifications that occur after the synthesis of protein. Proteins undergo different modifications such as phosphorylation, acetylation, ubiquitination, and so on. These modifications can alter the protein’s structure, function, and interaction, thereby regulating its biological activity. In neurodegenerative diseases, several proteins undergo abnormal post-translational modifications, which leads to aggregation and abnormal deposition of protein, thus resulting in neuronal death and related diseases. For example, the main pathological features of Alzheimer’s disease are the aggregation of beta-amyloid protein and abnormal phosphorylation of tau protein. The abnormal ubiquitination and loss of α-synuclein are related to the onset of Parkinson’s disease. Other neurodegenerative diseases such as Huntington’s disease, amyotrophic lateral sclerosis, and so on are also connected with abnormal PPTMs. Therefore, studying the abnormal PPTMs in neurodegenerative diseases is critical for understanding the mechanism of these diseases and the development of significant therapeutic strategies. This work reviews the implications of PPTMs in neurodegenerative diseases and discusses the relevant therapeutic strategies.

## Introduction

The development of human life science led to a deeper understanding of protein post-translational modifications (PPTMs). PPTMs play an important role in maintaining cellular homeostasis, signal transduction, metabolic regulation, and other biological processes in a cell. Disturbances in the balance of these modifications may trigger the occurrence of many diseases. Neurodegenerative disorders (NDDs) are a class of diseases characterized by neuronal damage and death, and among others commonly include Alzheimer’s Disease (AD), Parkinson’s Disease (PD), Huntington’s disease (HD), Amyotrophic Lateral Sclerosis (ALS), which bring great pain and burden to patients and their families. Interestingly, abnormal PPTMs are important causes of the occurrence and progression of these diseases. For example, abnormal phosphorylation of tau protein and ubiquitination imbalance of α-synuclein play a vital role in AD and PD respectively [1, 2]. Therefore, an in-depth study of the role and mechanism of PPTMs in neurodegenerative diseases will help us better understand the development of these diseases and provide new ideas and directions for better future treatment and prevention strategies.

**Fig. 1:**
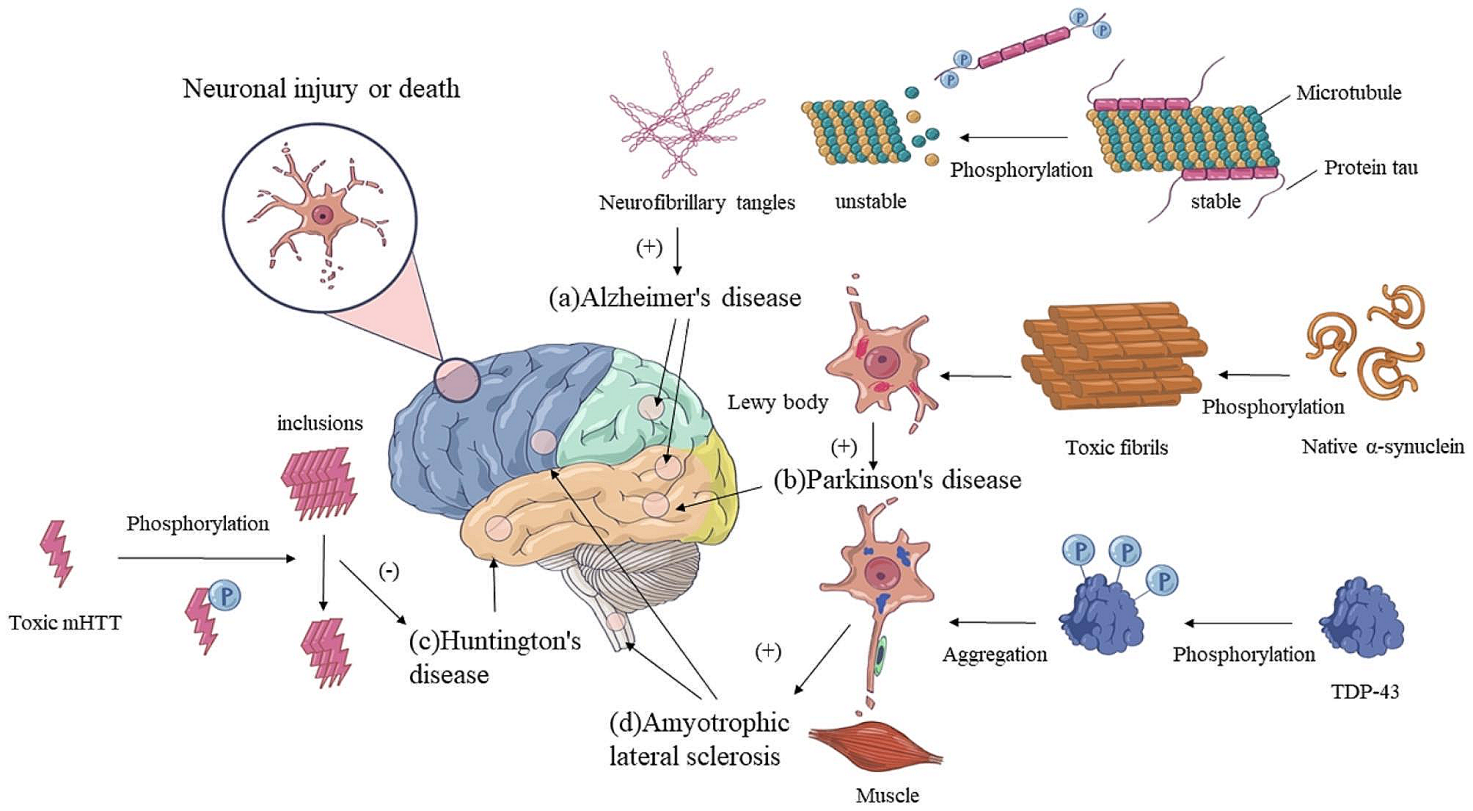
The role of PPTMs in neurodegenerative diseases. Take increased phosphorylation as an example: (a) AD: Phosphorylation affects the aggregation and stability of tau protein, which can lead to its abnormal aggregation and formation of neurofibrillary tangles, and ultimately the death of neurons [125]. (b) PD: Abnormal phosphorylation of α-synuclein may promote the formation of Lewy bodies in patients with Parkinson’s disease [126]. (c) HD: Toxic mHTT can be phosphorylated, and this can protect against the toxicity of polyQ-expanded HTT thus reducing neuronal damage [127]. (d) ALS: Phosphorylation of TDP-43 is one of the important factors leading to its abnormal aggregation. TDP-43 deposits exist in the brain and spinal cord of ALS patients as inclusion bodies, resulting in neuronal damage and death [128–134]

Phosphorylation is a common PPTM that can affect the activation, transport, and interactions of the affected protein. Phosphorylation influences synapse formation and function, resulting in impaired neuronal connection [3]. An important mechanism for regulating cell signal transduction is phosphorylation, which influences various aspects of neuronal functioning and neurobiological processes, such as the morphogenesis of neurons and the functions of synapses, glial cells, and mitochondria [4, 5]. Acetylation, a type of modification different from phosphorylation, can increase the stability and activity of a protein by changing its structure and properties [6–8]. Ubiquitination initiates a process of protein degradation that results in the breakdown of the tagged proteins and thus affects their stability [9–11]. By deubiquitinating Tau protein, the protein USP10 modulates its aggregation and thereby influences the pathogenesis of AD and other tauopathies, suggesting a possible therapeutic target for these neurodegenerative disorders [12]. The role of PPTMs in neurodegenerative diseases is briefly summarized in Fig. 1.

PPTMs play critical roles in the development as well as the progression of neurodegenerative diseases. Thus, a deep knowledge of these processes may provide a clear understanding of the molecular mechanisms of neurodegenerative diseases and give insight into better management strategies. We here provide an extensive literature review on PPTMs and their relation to neurodegenerative diseases and highlight the possible therapeutic strategies.

## Abnormal protein post-translational modifications and neurodegenerative diseases

### PPTMs and AD

Alzheimer’s disease is an age-related neurodegenerative disease, whose pathological features are the abnormal deposition of Aβ and Tau proteins. The abnormal deposition of Tau protein forms neurofibrillary tangles, which is one of the main pathological features of AD.

#### Phosphorylation and AD

Phosphorylation of proteins plays a significant role in AD. In AD, hyperphosphorylated tau protein accumulates as paired helical filaments that aggregate into masses inside nerve cell bodies known as neurofibrillary tangles. This is a common hallmark of neurodegenerative disorders, including AD.

In the early stages of tau processing in AD, the N-terminal part of the molecule undergoes a characteristic cascade of phosphorylation and progressive misfolding of the proteins [13]. This leads to a structural conformation detected by Alz-50. C-terminal truncation of tau at Asp-421 is an early event in tau aggregation [13]. Longfei Li et al. found that intra-cerebroventricular injection of 77G7 antibody (monoclonal antibodies) reduced tau levels in the wild-type FVB mouse brain and intravenous injection of 77G7 reduced tau hyperphosphorylation in the brain [14]. Using exosomes as a delivery system for curcumin, Hao Wang et al. demonstrate that this approach enhances the solubility and bioavailability of curcumin, facilitates its transport across the blood-brain barrier, and suppresses the abnormal phosphorylation of Tau protein by activating the AKT/GSK-3β pathway [15].

Oligomeric forms of Aβ were found to be abundant in synapses of AD patients early in the disease before the appearance of phospo-tau at later stages, suggesting that soluble Aβ oligomers in synaptic terminals are associated with dementia onset and may initiate a cascade that drives phosphorylated tau accumulation and its synaptic spread [16].

PP2A dysfunction has been linked to tau hyperphosphorylation, amyloidogenesis, and synaptic deficits, which are pathological hallmarks of AD [17]. In one study, synthetic tricyclic sulfonamides, which are PP2A activators, effectively increased PP2A activity, decreased tau phosphorylation, and Aβ_40/42_ levels in AD cell models [18].

Regarding the relationship between PPTMs and AD, there are already studies exploring mechanisms and treatment methods in this aspect. According to the findings of Daniel Giovinazzo et al., by sulfhydrating GSK3β and inhibiting its activity, hydrogen sulfide (H2S) prevents Tau hyperphosphorylation and enhances motor and cognitive functions in an AD mouse model [19].

A potential therapeutic option for AD by reducing p-p38 levels, alleviating microglia activation and amyloid-beta deposition, and improving spatial learning and memory in a 5xFAD transgenic mice model of AD was provided by PRZ-18,002, a protein degrader that selectively binds to an active form of p38 MAPK and induces its degradation, as found by Seung Hwan Son et al. [20].

#### Acetylation and AD

Ashutosh Tiwari’s team found that acetylation at lysine 16 led to the disordered aggregates that formed sticky but flexible amorphous structures and showed high levels of toxicity [21].

Decreased histone acetylation can lead to the downregulation of genes involved in synaptic plasticity, such as synaptophysin and BDNF, which are crucial for learning and memory [22–24].

We suspect that the increased proportion of acetylated tubulin in AD may represent an adaptive change in compensation for the loss of microtubules and their associated deficits in axonal transport along microtubules.

Fan Zhang et al. hypothesize that the rise in acetylated tubulin levels in AD could be an adaptive response to compensate for the reduction in microtubules and the resulting impairments in axonal transport along these microtubules [25].

#### Ubiquitination and AD

Ubiquitination has been implicated in the pathogenesis of AD. The ubiquitin proteasome system (UPS) clears the cell of dysfunctional and misfolded proteins via ubiquitination and degradation. Amyloid-β peptide, the main constituent of plaques in AD, has been shown to inhibit 26 S proteasome activity, thus exacerbating ubiquitin-dependent degradation [26]. Treadmill exercise ameliorated Alzheimer’s disease-associated cognitive dysfunction, amyloid plaque pathology, Tau hyperphosphorylation, and ubiquitin–proteasome system dysfunction in APP/PS1 transgenic mice [27]. Disruption of endosomal sorting complexes required for transport (ESCRT) -mediated APP trafficking and ubiquitination by ubiquitination factor E4B (UBE4B) dysregulation contributes to Aβ accumulation in Alzheimer’s disease [28]. Tetramethylpyrazine ameliorates Alzheimer’s disease progression by mitigating neuronal apoptosis and injury through CUL4B-mediated suppression of SSTR4 ubiquitination [29]. RBFOX1 ubiquitination is increased in Alzheimer’s disease brain tissue, particularly in axons, and this increase is partly mediated by adenosine 2a receptor(A2AR) signaling [30]. Nicha Puangmalai et al. demonstrate that K63-linked ubiquitination of soluble tau oligomers (TauO) enhances its seeding activity and propagation, suggesting a novel therapeutic target for Alzheimer’s disease and related tauopathies. BACE1 SUMOylation is a reciprocal regulator of its phosphorylation and ubiquitination, which may have implications for the regulation of BACE1 activity and Aβ accumulation in Alzheimer’s disease [31]. N6-methyladenosine(m6A)-modified circRIMS2 contributes to synaptic and memory impairments in AD by regulating GluN2B ubiquitination [32].

#### The mechanism of key protein truncation involved in AD

Tau protein truncation is a key mechanism in the pathogenesis of Alzheimer’s disease (AD) [33]. Tau is a microtubule-associated protein that plays a crucial role in stabilizing the cytoskeleton of neurons [34]. There are several mechanisms by which tau truncation can occur in AD. One mechanism is through proteolytic cleavage by caspases, which are enzymes that are activated during apoptosis (programmed cell death) [35, 36]. Caspases can cleave tau at several different sites, resulting in the formation of truncated tau fragments of varying lengths [37]. Another mechanism of tau truncation is through alternative splicing of the tau gene. Alternative splicing is a process by which different exons of a gene can be spliced together to produce different isoforms of a protein [38]. Truncated tau fragments are more toxic than full-length tau. They are more likely to aggregate and form NFTs, and they can also disrupt the normal function of other proteins in the neuron [39]. Overall, tau truncation is a key mechanism in the pathogenesis of AD. Truncated tau fragments are more toxic than full-length tau and can contribute to neuronal dysfunction, death, and the spread of tau pathology. According to Henriette Haukedal et al., Golgi fragmentation is an early disease phenotype in AD neurons that can be aggravated by additional risk variants in SORL1 [40].

PPTMs may play an important role in the occurrence and development of AD, but figuring out their specific implication mechanisms needs further studies. In the future, researchers need to explore the relationship between protein modifications and AD more deeply to find more effective treatments, thus providing a better quality of life for AD patients.

### PPTMs and PD

PD is one of the common chronic progressive neurodegenerative diseases, whose pathogenesis is still not fully understood. A growing body of research evidence indicates that the progression of PD is strongly associated with the presence of PPTMs.

#### Phosphorylation and PD

Using cryo-electron microscopy (cryo-EM), Kun Zhao et al. elucidated the structure of the α-synuclein amyloid fibril and revealed that phosphorylation at Tyr39 induces the formation of polymorphic fibrils with a markedly enlarged fibril core [41]. In their study, Farzaneh Atashrazm et al. examined the phosphorylation of a novel LRRK2 substrate, Rab10, to assess its suitability as a target engagement biomarker and/or a patient enrichment biomarker for clinical trials involving LRRK2 inhibitors [42]. They discovered that phosphorylation of Rab10 at threonine 73 was a valid indicator of target engagement, but its use as a patient enrichment biomarker was hindered by the lack of correlation between LRRK2 and Rab10 phosphorylation [42]. Moreover, they found that neutrophils from PD patients had elevated levels of LRRK2, which could be useful for patient stratification, and that LRRK2 activity in peripheral immune cells might be involved in an inflammatory phenotype [42]. Shijie Wang et al. examined the phosphorylation of Rab10, a novel substrate of LRRK2, as a possible target engagement and/or patient enrichment biomarker for clinical trials of LRRK2 inhibitors, and concluded that phosphorylation of Rab10 at T73 is a valid target engagement biomarker, but its use as a patient enrichment biomarker is hindered by the lack of correlation between LRRK2 and Rab10 phosphorylation, and that elevated LRRK2 levels in PD patients’ neutrophils may be useful for patient stratification and LRRK2 activity in peripheral immune cells may contribute to an inflammatory phenotype [43]. Using a mouse model of PD, Kentaro Togashi et al. explored the impact of phosphorylation of Collapsin Response Mediator Protein 2 (CRMP2) on PD pathogenesis and found that genetically inhibiting CRMP2 phosphorylation ameliorated the condition of mice exposed to a PD-inducing neurotoxin [44]. This implies that targeting CRMP2 phosphorylation could be a new therapeutic strategy for PD [44]. Jia Liu et al. studied the beneficial effects of piperlongumine (PLG) in rotenone-induced PD cell and mouse models [45]. They found that PLG administration reduced motor deficits, increased cell viability, and enhanced mitochondrial function. PLG exerted its beneficial effects by preventing apoptosis and inducing autophagy, which was mediated by enhanced phosphorylation of BCL2 at Ser70 [45]. These results that c-Abl and PHB2 may serve as potential therapeutic targets for the treatment of PD [46]. Using imaging mass cytometry to analyze postmortem human brain sections from patients with PD, Chun Chen et al. found that astrocytes, like neurons, are vulnerable to mitochondrial defects, which could affect their reactivity and ability to support neurons in PD [47]. The study also revealed deficiencies in respiratory chain subunit expression within astrocytes and changes associated with PD that are not solely due to advancing age.

#### Acetylation and PD

Acetylation also plays an important role in PD. Ian F. Harrison et al. discovered that PD is characterized by the degeneration of nigrostriatal neurons, resulting from intracytoplasmic inclusions primarily composed of α-synuclein, a synaptic protein [48]. They further demonstrated that the imbalance between histone acetyltransferases (HATs) and HDACs can be corrected by using HDAC inhibitors, which can help regulate transcription, maintain neuronal homeostasis, and provide neuroprotection in disorders such as PD [48]. Goonho Park et al. discovered that PD-related neurotoxins trigger histone acetylation and HDAC degradation via autophagy, resulting in increased histone acetylation levels in dopaminergic neurons, which may play a role in PD pathogenesis [49]. As a result of 3,4-dihydroxyphenylacetaldehyde (DOPAL), Vanderlei de Araujo Lima and colleagues investigated how N-terminal acetylation and familial Parkinson’s disease-linked mutations affected oligomerization of a-synuclein [50]. The N-terminal acetylation reduces DOPAL-induced oligomer formation, but the familial mutation increases it [50]. By conducting a genome-wide histone acetylation analysis, researchers discovered altered transcriptional regulation in the PD brain, indicating that aberrant histone acetylation and transcriptional regulation contribute to the pathophysiology of PD [51]. Furthermore, the study revealed that PD-associated genes are particularly susceptible to epigenetic dysregulation [51]. Hydrogen sulfide was found to exert a protective effect against Parkinson’s disease by inhibiting epigenetic histone acetylation in a rat model induced with 6-hydroxydopamine, using sodium hydrogen sulfide as a hydrogen sulfide donor [52]. Fei Fan et al. found that the nuclear translocation of PGC-1α, mediated by acetylation and phosphorylation, protects against oxidative damage in an MPP+-induced cell model of Parkinson’s disease, suggesting that therapeutic reagents activating PGC-1α may be valuable for preventing mitochondrial dysfunction in PD by mitigating oxidative damage [53]. In their research, Rohan Gupta and Pravir Kumar explore the role of PARP1 as an interactor of histone deacetylases, sharing common lysine residues for acetylation, ubiquitination, and SUMOylation in Alzheimer’s and Parkinson’s disease, and conclude that the loss of acetylated hotspot sites results in the loss of ubiquitination and SUMOylation function on nearby sites [54]. The newly synthesized HDAC6 inhibitor, HGC, was discovered to rescue dopaminergic neuron loss in PD models by inducing acetylation of NDUFV1, thus demonstrating significant therapeutic potential for treating PD [55]. In their investigation of the DOPAL-induced oligomerization of α-synuclein (aSyn), Vanderlei de Araújo Lima et al. discovered that N-terminal acetylation significantly reduces the formation of DOPAL-induced aSyn oligomers, while familial mutations increase it [56]. Additionally, they found that the binding of aSyn to phospholipid vesicles hinders the formation of DOPAL-mediated aSyn oligomers [56].

#### Ubiquitination and PD

One of the widely studied modifications is ubiquitination. A new 22-kilodalton glycosylated form of alpha-synuclein, which is ubiquitinated by parkin, an E3 ubiquitin ligase, in normal human brain, was identified by Shimura, Hideki et al., and they proposed that this reaction prevents pathological alpha-synuclein accumulation, which occurs due to loss of parkin function and may contribute to the ubiquitinated alpha-synuclein accumulation in PD [57]. According to a study by Matthew E. Gegg and Anthony H.V. Schapira, parkin expression can restore the impaired autophagic flux and mitochondrial function that are associated with PINK1 deficiency in human dopaminergic cells [58]. The process of PINK1-parkin-dependent mitophagy is further analyzed to reveal that the early event is the ubiquitination of mitofusins 1 and 2 [58]. By preventing parkin from functioning as a ubiquitin E3 ligase through S-nitrosylation, these disorders could accelerate the degeneration process by hindering the ubiquitination of parkin substrates [59].

#### Palmitoylation and PD

By comparing the cerebral cortex of PD patients and controls, Juan F. Cervilla-Martínez et al. identified 150 proteins with different levels of palmitoylation, a post-translational modification that may be involved in the pathophysiology of PD. This study demonstrates the potential of comprehensive palmitoyl-proteomics to reveal novel cellular mechanisms affected by this neurodegenerative disease [60].

#### Adenylation and PD

According to a study by Anwesha Sanyal et al., HYPE, which is a Fic protein in humans, can adenylylate Alpha-Synuclein, a protein implicated in PD. This adenylation process reduces the aggregation-related phenotypes of Alpha-Synuclein, implying a cellular mechanism to cope with its toxicity [61].

#### Glycosylation and PD

Glycosylation is an important post-translational modification of proteins, which has been proven to be related to the pathogenesis of PD. Csaba Váradi et al. have introduced a novel capillary electrophoresis technique, coupled with label-free quantitation and support vector machine-based feature selection, for identifying potential glycan alterations in PD [62]. The study detected reduced sialylation and elevated fucosylation in PD patients on tri-antennary glycans with 2 and 3-terminal sialic acids [62]. Moreover, the method was effective in accurately classifying male patients [62]. Nicholas P. Marotta’s research indicates that the modification of α-Synuclein by O-GlcNAc significantly suppresses aggregate formation and toxicity in neurons [63]. These findings suggest that O-GlcNAc modification and the enzymes involved in its regulation hold potential as therapeutic targets in the quest for improved Parkinson’s disease treatments.

#### Oxidation and PD

Oxidative modification can affect various aspects of the pathogenesis of PD. Hector Flavio Ortega-Arellano et al. found that exposing Drosophila melanogaster to polyphenols extended their lifespan and restored their ability to move when chronically exposed to paraquat, compared to flies treated with paraquat alone in 1% glucose or 10% glucose partially prolonged the lifespan and climbing ability of Drosophila exposed to iron, PQ or a combination of both, with the investigation being the first to report that Ddc-GAL4 transgenic flies fed with polyphenols chronically increase their lifespan and enhance their movement abilities compared to untreated Ddc-GAL4 or those treated with paraquat in 1% glucose, supporting the idea that Drosophila melanogaster can serve as a suitable model to study genetic, environmental, and nutritional factors that may cause or modulate the development of PD [64]. Researchers used a novel quantitative mass spectrometry approach to measure relative changes in oxidation at specific sites in mutant DJ-1 compared to the wild-type protein and found that the M26I familial substitution and methionine oxidation characteristic of sporadic PD may disrupt DJ-1 function by disfavoring a site-specific modification required for optimal neuroprotective activity, indicating the effect of a single amino acid substitution on oxidative modifications of the PD-related protein DJ-1 [65].

#### The mechanism of key protein truncation involved in PD

Protein truncation has been implicated in the pathogenesis of PD. Alpha-synuclein is a protein that is normally found in the presynaptic terminals of neurons [66]. Truncation or incomplete proteolysis of α-synuclein causes Parkinsonism through the aggregation of the truncated species [67]. A novel method enables the production of pure N-terminally truncated α-synuclein, revealing the crucial role of its first six residues in amyloid formation [68].

PPTMs are closely related to the pathogenesis of PD and different types of modification may affect the structure and function of proteins through different pathways, which result in the development of PD. Therefore, studying the effects of these modifications in PD may further provide new ideas and methods for developing new treatments and preventive measures.

### PPTMs and HD

HD, a genetic neurodegenerative disease, is thought to be associated with abnormal modifications of huntingtin protein (HTT), which may play a critical role in the pathogenesis of HD.

#### Phosphorylation and HD

Phosphorylation of HTT is an important form of modification in HD. Isaline Mees et al. examined alterations in the cortical phosphoproteome of 8-week-old and 28-week-old R6/1 transgenic HD mice and observed significant protein phosphorylation dysregulation in the cerebral cortex of HD mice before the onset of symptoms [69]. Cristina Cariulo et al. discovered that phosphorylation on residue T3 in the N17 region of the HTT, a posttranslational modification, can reverse the conformational effects of the HD mutation on the HTT and inhibit its aggregation properties in vitro, offering insights into mechanisms of HD pathogenesis and creating new prospects for the development of therapeutics and diagnostics for HD [70]. Veronica Brito et al. demonstrated that CDK5 dysfunction contributes to depressive-like behaviors in HD by altering the theDARPP-32 phosphorylation status in the Nucleus Accumbens and that CDK5 inhibition prevented depressive-like behavior and reduced DARPP-32 phosphorylation, indicating that CDK5 is a crucial factor in the development of depressive-like behaviors in HD mice [71]. Mijung Lee et al. discovered that Beta-Lapachone improves HD phenotypes by increasing Sirt1 expression, CREB phosphorylation, and PGC-1α deacetylation, with oral administration of Beta-Lapachone to R6/2 HD mice resulting in enhancements in behavioral phenotypes related to HD, such as impairment of rota-rod performance and increase of clasping behavior, as well as changes in Sirt1 expression, CREB phosphorylation, and PGC-1α deacetylation, highlighting Beta-Lapachone’s potential as a therapeutic candidate for the treatment of HD-associated phenotypes [72].

#### Acetylation and HD

Leah Gottlieb et al. discovered that N-alpha-acetylation of HTT increases its propensity to aggregate, which has implications for HD since aggregation of HTT is associated with the disease, and the study suggests that targeting NatA-mediated Htt acetylation could be a promising therapeutic approach in HD [73]. Adding acyl groups (such as acetyl groups) to protein molecules through acylation modification is a means of regulating protein function. Inhibiting Histone Deacetylase 6 (HDAC6) compensates for the transport deficit in HD by increasing tubulin acetylation, and HDAC inhibitors enhance the vesicular transport of brain-derived neurotrophic factor (BDNF) by inhibiting HDAC6, which raises acetylation at lysine 48 of a-tubulin, thereby increasing the flux of vesicles and the subsequent release of BDNF, indicating that HDAC6 inhibition and acetylation at lysine 40 of a-tubulin could be promising therapeutic targets for disorders such as HD in which intracellular transport is disrupted [74]. Dietary restriction (DR) rectified several effects of the transgene in the full-length YAC128 HD mouse model, such as increased body weight, reduced blood glucose, and impaired motor function, with these changes linked to decreased striatal human (but not mouse) HTT expression and changes in gene expression regulating histone acetylation modifications, particularly Hdac2, indicating a protective effect of DR in a transgenic model containing the complete human HTT gene, and for the first time suggesting a role for DR in reducing HTT level, which correlates with symptom severity [75]. Scientists utilized mice in which SIRT2 has been reduced or ablated to investigate the function of SIRT2 and determine whether SIRT2 loss has a beneficial impact on disease progression in the R6/2 mouse model of HD and found that SIRT2 ablation did not affect tubulin acetylation in the brain, cholesterol biosynthesis, or the progression of HD phenotypes in vivo, as assessed by a battery of physiological and behavioral tests [76]. Scientists discovered that dysfunction of upstream binding factor-1 (UBF-1) is associated with decreased ribosomal DNA (rDNA) transcription in HD, with UBF1 acetylation at Lys (K) 352 by CREB binding protein (CBP) crucial for the transcriptional activity of rDNA, and abnormal activity of UBF1 and its acetylation by CBP linked to impaired rDNA transcription in HD, indicating that modulation of UBF-mediated rDNA synthesis by CBP could be a promising therapeutic target for enhancing neuronal rDNA transcription in HD [77]. In the R6/2 mouse model of HD, genetic depletion of HDAC6 led to a significant increase in tubulin acetylation throughout the brain, but did not impact the onset and progression of various behavioral, physiological, molecular, and pathological HD-related phenotypes, nor did it affect the aggregate load or levels of soluble mutant exon 1 transprotein or the efficiency of BDNF transport from the cortex to the striatum, indicating that HDAC6 inhibition does not alter disease progression in R6/2 mice and should not be prioritized as a therapeutic target for HD [78].

#### Ubiquitination and HD

Abnormal aggregation of HTT is a major pathological feature of HD, with ubiquitination being an important mechanism for HTT degradation, and Nelma M. Palminha et al. discovered that defective repair of topoisomerase I induced chromosomal damage in HD is due to reduced H2A ubiquitination resulting from limited RNF168 activity, which is caused by increased interaction with p62, and that depletion of p62 or disruption of the interaction between RNF168 and p62 is sufficient to restore 53BP1 enrichment and subsequent DNA repair in HD models, presenting new possibilities for therapeutic interventions [79]. Siah-1-interacting protein (SIP) was found by Ewelina Latoszek et al. to regulate mutated HTT aggregation in HD models, with an increase in SIP dimerization in HD medium spiny neurons causing a decrease in SIP function in the degradation of mHTT via a ubiquitin-proteasome pathway, increasing mHTT aggregation, indicating that SIP may be a potential target for anti-HD therapy during the early stage of HD pathology [80]. Ubiquitin conjugation to target proteins followed by their proteasomal degradation has become the hallmark of the mechanism by which cells remove regulatory proteins, tune their activity, or destroy damaged proteins, as discovered by Noam E. Ziv and Aaron Ciechanover, who also described another nonproteolytic role of ubiquitin modification in attenuating the pathogenic effects of polyQ-expanded aggregate-prone HTT, the protein responsible for HD [81]. Anna Pluciennik et al. discovered that the deubiquitinase USP7 selectively interacts with polyQ-expanded androgen receptor (AR) in spinal and bulbar muscular atrophy (SBMA) and that decreasing usP7 levels reduced mutant AR aggregation and cytotoxicity in cell models of SBMA, suppressed disease phenotypes in SBMA and spinocerebellar ataxia type 3 (SCA3) fly models, and improved several motor deficiencies in transgenic SBMA mice, highlighting the critical role of USP7 in the pathophysiology of SBMA and suggesting a similar role in SCA3 and HD [82]. Using label-free quantitative mass spectrometry techniques, Karen A. Sap et al. identified proteome and ubiquitinome alterations in brain tissue of HD mouse model Q175FDN and wild-type mice, revealing differential ubiquitination of wild-type and mutant Huntingtin in both Triton X-100 soluble and insoluble fractions, and indicating that the disease affects cellular processes such as vesicular transport, gene expression, translation, catabolic processes, and oxidative phosphorylation [83]. Huanhuan Luo et al. discovered that Homocysteine-induced endoplasmic reticulum protein (Herp) can bind to overexpressed huntingtin N-terminal, enhance its ubiquitination, and reduce its cytotoxicity, indicating that Herp is a newly identified huntingtin-interacting protein that can decrease the cytotoxicity of mutant huntingtin by inhibiting its aggregation and promoting its degradation [84]. Jordan J. S. VerPlank et al. discovered that cGMP, through PKG, stimulates 26 S proteasomes and augments the degradation of proteins, including those responsible for neurodegenerative diseases, indicating that compounds that elevate cGMP levels could potentially slow the progression of neurodegenerative diseases by stimulating cytosolic proteasomes, protein ubiquitination, and overall protein degradation [85]. Mali Jiang et al. discovered that Nemo-like kinase (NLK) interacts with HTT and that NLK levels are significantly reduced in HD human brain and HD models, demonstrating that NLK overexpression in the striatum reduces brain atrophy and mutant HTT(mHTT) aggregation in HD mice, lowers mHTT levels in a kinase activity-dependent manner while having no significant effect on normal HTT protein levels, and promotes mHTT ubiquitination and degradation via the proteasome pathway, suggesting a protective role of NLK in HD and identifying a new molecular target to reduce mHTT levels [86].

#### Palmitoylation and HD

Fanny L. Lemarié et al. reveals that HTT is palmitoylated at multiple sites and post-translationally myristoylated following caspase cleavage, and that blocking caspase cleavage at the critical and pathogenic aspartate 586 significantly increases posttranslational myristoylation of huntingtin, promoting the co-interaction between C-terminal and N-terminal huntingtin fragments and providing a protective effect [87].

From the data summarized above, it is clear that abnormal PPTMs of HTT protein play an important role in the pathogenesis of HD. These forms of modification may interact with each other and thus might exacerbate the condition. Future investigations are needed to explore the mechanisms of these forms of modifications more deeply as well as how their interactions might affect the disease to provide new ideas and methods for the management of HD.

### PPTMs and ALS

ALS is a type of degenerative disease of the nervous system, which is characterized by the death of neurons leading to muscle atrophy. Accumulating studies indicate that abnormal protein modifications both inside and outside cells are involved in the pathogenesis of ALS.

#### Phosphorylation and ALS

Phosphorylation modification is an important mechanism in cell signaling pathways that can regulate biological processes such as cell growth, proliferation, differentiation, and apoptosis. The drug Riluzole, commonly used to treat ALS, has been discovered to have micromolar inhibitory activity towards protein kinase CK1δ, indicating a link between its action and the inhibition of CK1δ, which prevents TDP-43 hyperphosphorylation, and presenting new opportunities for the development of more potent treatments for ALS and other TDP-43 related proteinopathies [88]. Novel genetic variants in MAPT and changes in tau phosphorylation in ALS post-mortem motor cortex and cerebrospinal fluid have been identified by Tiziana Petrozziello et al., indicating the potential involvement of tau phosphorylation in ALS and proposing that cerebrospinal fluid total tau and pTau-T181 ratio could be used as biomarkers for ALS [89].

#### Acetylation and ALS

Archana Prasad et al. discovered that kosmotropic anions significantly accelerate the aggregation of a C-terminal region of TDP-43 implicated in ALS, while chaotropic anions hinder it, that acetylation of specific lysines in C-terminal fragments considerably reduces the TDP-432 C’s amyloid-like aggregation, and that spontaneously formed cysteine-linked homodimers of the recombinantly purified TDP-432 C maintain amyloidogenicity [90]. Dong Liu et al. performed a proteomic analysis of post-mortem spinal cord tissues from individuals with and without ALS, revealing differential regulation of protein acetylation between the two groups, with GFAP being highly acetylated in ALS spinal cord and its larger fragments being upregulated in ALS spinal cord, indicating that acetylation and/or deacetylation may have a significant role in the development of ALS [91].

#### Ubiquitination and ALS

Before their death and disappearance, motoneurons in ALS-SOD1 mice undergo a prolonged sick phase, marked by the gradual buildup of ubiquitinated material.

#### Glycosylation and ALS

Glycosylation modification is a chemical reaction between sugars and proteins, which can change the structure and function of proteins. Xiaoyang Shan et al. discovered reduced protein O-glycosylation in the nervous system of the mutant SOD1 transgenic mouse model of ALS, with decreased O-GlcNAc immunoreactivity levels in spinal cord tissue from mSOD mice compared to controls, indicating that the neurodegeneration observed in mSOD mice is linked to a decrease in O-GlcNAc levels in neurons, including motor neurons [92].

Overall, abnormal PPTMs may be one of the most important factors contributing to ALS. Modifications such as phosphorylation, glycosylation, and oxidation as well as gene expression changes via altered acetylation may all be related to neuronal degeneration and inflammation, and thus play important roles in the development of ALS. Therefore, studying these abnormal modifications may help further understand the pathogenesis of ALS and provide new insights into its diagnosis and treatment.

## Treatment strategy based on PPTMs

Neurodegenerative diseases including AD, PD, HD, and ALS are one of the heaviest burdens of neurological disorders especially among the elderly population. Therefore, the is a need for significant and effective therapeutic strategies for their management. Interventions based on the pathogenic processes of neurodegenerative diseases are the main strategies of prevention and treatment. Abnormal PPTMs are key pathogenic mechanisms of neurodegenerative diseases. Thus, a better understanding of PPTMs might provide a clue in their management. There have been a series of advances in drug screening and development that target abnormal PPTMs in several neurodegenerative diseases.

Yuyou Zhu and Juan wang identified wogonin, a natural product that can effectively enhance Ap clearance in primary neural astrocytes and significantly reduce Ap secretion in SH-SY5Y-APP and BACE1cells through the mTOR/autophagy signaling pathway, while also inhibiting the activity of GSK3b via mTOR inhibition, ultimately resulting in a decrease in tau phosphorylation in SH-SHY5Y cells and primary neural astrocytes, demonstrating its potential as a therapeutic agent for treating AD [93]. Guiliang Zhang et al. demonstrated that T-806, a small-molecule compound derived from tetramethylpyrazine (TMP), enhanced cognitive function in AD mouse models by decreasing p-tau and total tau levels and increasing the expression of synapse-associated proteins [94]. Lei Zhu et al. discovered that pseudo-ginsenoside-F11 (PF11) improved cognitive deficits in a rat model of sporadic Alzheimer’s disease in a dose-dependent manner by regulating the insulin signaling pathway and calpain I/CDK5 signaling pathway in the hippocampus [95]. Barbara Bettegazzi et al. observed that neuronal activity stimulates Casein Kinase 2-dependent phosphorylation of the translation initiation factor eIF4B, which subsequently regulates BACE1 expression and APP processing, explaining the connection between brain activity and amyloid production and deposition in AD [96]. Meeting Li et al. demonstrated that genipin could significantly decrease phosphorylated Tau level and Aβ generation in vitro by binding to Tau, reducing the expression of CDK5 and GSK-3β, activating mTOR-dependent autophagy through the SIRT1/LKB1/AMPK signaling pathway in Tau-overexpressing cells, and suppressing BACE1 expression via the PERK/eIF2α signaling pathway in N2a/SweAPP cells [97]. Ana M. de Matos et al. discovered that glucosyl polyphenols show promise as inhibitors of Aβ-induced Fyn kinase activation and Tau phosphorylation in AD and type 2 diabetes, with several compounds found to inhibit Aβ-induced Fyn kinase activation and reduce pTau levels [98]. Beta boswellic acid, a primary component of the Boswellia serrata plant, exhibits a protective effect against streptozotocin-induced sporadic AD by reducing tau hyperphosphorylation, increasing reelin levels, and enhancing learning and memory in rats [99]. Cinnamaldehyde (Cin), a primary compound of cinnamon, ameliorated recognition/spatial memory impairments and anxiety-like behavior, reversed STZ-induced effects on AB aggregation and caspase-3 cleavage in the hippocampus, and regulated hippocampal IRS-1/AKT/GSK-33 phosphorylation in a model of sporadic Alzheimer’s disease [100]. Pioglitazone, a PPARγ agonist, can decrease β Amyloid levels in a neuronal model of AD by suppressing PPARγ phosphorylation and reducing CDK5 expression [101]. Tripterygium glycoside (TG) improves spatial memory and learning abilities, reduces the expression of AB25-35, p-Tau, and CD11b, increases neuron density, and suppresses the release of inflammatory factors and microglial activity by inhibiting the phosphorylation of IκBα and p38 MAPK, thus alleviating neuroinflammation in a mouse model of Aβ25-35-induced AD [102]. Pimavanserin restored normal locomotion in both the P381L/COMT- and rTg(P301L)4510 mouse models of AD, without affecting sensorimotor gating or tau phosphorylation patterns, indicating that pimavanserin may alleviate excessive locomotion driven by tau [103]. Hoon Lim et al. explored the use of human umbilical cord blood-derived mesenchymal stem cells (hUCB-MSCs) to inhibit tau hyperphosphorylation and discovered that hUCB-MSCs can alleviate tau hyperphosphorylation by secreting GAL-3, revealing their potential as a therapeutic agent for abnormal tau in AD [104]. N6-Furfuryladenine (N6FFA) exerts a protective effect in HD models by promoting huntingtin phosphorylation and has the potential to reverse disease phenotypes and decrease mutant huntingtin levels [105]. Although Rab10 T73 phosphorylation is a promising target engagement biomarker for potential use in leucine-rich repeat kinase 2 (LRRK2) inhibitor clinical trials, its potential use as a patient enrichment biomarker is complicated by the absence of a correlation between LRRK2 and Rab10 phosphorylation [42]. 2-aminoquinoline attenuated motor deficits and suppressed MPP+-induced astrocyte apoptosis in a mouse model of MPTP-induced PD by regulating the Bax/Bcl-2 ratio via targeting p-JNK [106].

Maria Nguyen and Dimitri Krainc found that LRRK2 phosphorylation of auxilin causes synaptic impairments in dopaminergic neurons from PD patients and that restoring auxilin function in mutant LRRK2 dopaminergic neurons partially alleviates pathogenic phenotypes [107]. Marwa E. A. El-Shamarka demonstrates that inosine protects against rotenone-induced PD in mice by mitigating neuroinflammation and oxido-nitrosative stress, inhibiting ERK phosphorylation, and reducing A2AR expression [108]. Riluzole, a medication utilized in the treatment of ALS, exhibits micromolar inhibitory activity against protein kinase CK1δ, which connects the two primary clinical symptoms of ALS: glutamate-mediated excitotoxicity and TDP-43 related proteinopathy [109]. Integrative analysis of multi-omic data and validation using a yeast model revealed that oxidative phosphorylation regulates protein aggregation in ALS, highlighting the crucial involvement of mitochondrial oxidative phosphorylation in amyloidogenesis and its potential as a therapeutic target in ALS [110].

Treatment with the HDAC inhibitor MS-275 and theAMPK/sirtuin 1 activator resveratrol normalized RelA acetylation in sOD1(G93A) mice, delayed disease onset, enhanced motor function, prolonged lifespan, and rescued lumbar motor neurons affected in sOD1(693 A) mice [111]. Trichostatin A, a histone deacetylase inhibitor, alleviates growth inhibition and restores histone acetylation at specific sites in a FUS ALS/FTD yeast model, offering a new mechanistic basis for alternative therapeutic strategies in ALS/FTD and other neurodegenerative disorders [112]. Acetylation (neutralization) of multiple lysines by aspirin can increase the intrinsic net negative charge of the polypeptide and inhibit the amyloidogenesis of superoxide dismutase (SOD1) [113]. Delayed-start administration of the HDAC inhibitor sodium valproate in a lactacystin rat model of PD resulted in dose-dependent neuroprotection/restoration against lactacystin neurotoxicity, as evidenced by the alleviation of motor deficits, attenuation of morphological brain changes, and restoration of dopaminergic neurons in the substantia nigra [114]. HGC, a novel HDAC inhibitor, protects dopaminergic neurons from MPP+-induced damage and improves symptoms in an MPTP-induced PD mouse model by inhibiting HDAC6 and promoting acetylation of NDUFV1, an enzyme in the electron transport chain complex I, indicating that HGC may have therapeutic potential for PD treatment [55]. Neither genetic reduction nor ablation of SIRT2 affected the acetylation of alpha-tubulin or H4K16 or cholesterol biosynthesis in the brains of wild-type mice, nor did it alter HD progression, aggregate load, or levels of soluble mutant huntingtin transporting, and neither constitutive genetic loss nor acute pharmacological inhibition of SIRT2 affected the expression of cholesterol biosynthesis enzymes in the context of HD, indicating that SIRT2 inhibition is not a viable therapeutic option for HD [76]. The significant overlap between genes regulated by the histone deacetylase inhibitor TSA and differentially expressed genes in the AD brain suggests that epigenetic mechanisms in neurons may be involved in the early stages of AD [115]. Acetylation of Transcription factor EB (TFEB) facilitates its nuclear translocation and lysosome biogenesis, independent of TFEB dephosphorylation, and treatment with TSA, an inhibitor of HDACs, upregulates the expression of lysosomal genes, enhances Aβ aggregate clearance in APP/PS1 mouse brains, and improves their memory, highlighting the potential of HDAC inhibition to promote lysosome biogenesis and serve as a therapeutic strategy for neurodegenerative diseases [116]. Aberrant expression of p75 in a rotenone-based stereotactic infusion in vivo model of PD led to significant upregulation of siAH compared to the control group, and in cellular models of rotenone-mediated neurotoxicity, p75 was found to interact with siAH through immunoprecipitation, promoting nuclear expression of NF-jB (p65) that may interact with the promoter of the siAH gene, while siRNA-mediated p75 depletion reduced the upregulation of a-syn and nuclear expression of p65, and protected against rotenone-induced cell apoptosis, suggesting that p75 modulates the increased expression of a-syn, which is associated with siAH-mediated ubiquitination and nuclear expression of p65 [117]. The CDK5-GP78 pathway plays a role in the pathogenesis of PD and may be a potential drug target for its treatment, as demonstrated by the protective effects of overexpressing GP78 or interfering with GP78 Ser516 phosphorylation against MPP+-induced cell death in neurons [118].

Yongchang Diwu et al. demonstrated that Xixin decoction (XXD) can enhance O-GlcNAc glycosylation of tau proteins in the hippocampus of rats with sporadic Alzheimer’s disease (SAD), which may inhibit hyperphosphorylation of tau proteins on key sites, reduce their toxicity, and prevent the pathological process of SAD [119]. The intensity and modulation of antioxidant, anti-AD, anti-diabetic, and anti-inflammatory effects of luteolin and its C-glycosylated derivatives may be closely linked to C-glycosylation at different positions, as discovered by Jae Sue Choi et al. [120]. Apigenin and its two C-glycosylated derivatives, vitexin, and isovitexin, exhibit strong anti-diabetic, anti-AD, and anti-inflammatory properties, with isovitexin demonstrating the most potent inhibitory effects against rat lens aldose reductase, human recombinant aldose reductase, advanced glycation endproducts, acetylcholinesterase, and butyrylcholinesterase, while vitexin displayed the most potent protein tyrosine phosphatase 1B inhibitory activity, and apigenin exhibited potent anti-inflammatory activity by suppressing nitric oxide production and expression of inducible nitric oxide synthase and cyclooxygenase-2 [121].

HDAC1 SUMOylation serves as a natural defense mechanism against Aβ toxicity, with acute Aβ inducing the expression of PIAS1 and Mcl-1 through MAPK/ERK activation, PIAS1 enhancing HDAC1 SUMOylation in the rat hippocampus, reducing the association of sumoylated HDAC1 with CREB, increasing CREB binding to the Mcl-1 promoter, and mediating Aβ-induced Mcl-1 expression, while transduction of a SUMO-modified lenti-HDAC1 vector into the hippocampus of APP/PS1 mice rescued spatial learning and memory deficits, improved long-term potentiation impairment, and reduced amyloid plaque and apoptotic cells in the CA1 area [122].

Several potential therapeutic strategies could be used to target tau truncation in AD. One approach is to develop caspase inhibitors that can block the proteolytic cleavage of tau [123]. Additionally, Valentina Latina et al. have developed a monoclonal tau antibody (12A12mAb) that can specifically target truncated tau fragments [124].

Numerous studies have identified several potential treatment strategies for prion diseases, such as prion protein inhibitors, mitochondrial protectors, vitamin D supplements, RNA interference, optogenetic techniques, immunotherapy, and cell therapy.

## Conclusion

In regulating numerous cellular processes, post-translational modification of proteins is a crucial mechanism. However, alterations of PTMs might be implicated in the pathological mechanism of neurodegenerative and other diseases. Targeting PTMs represents an important therapeutic strategy for the management of these disorders. In this regard, many products have been proposed however more research is needed to further explore the molecular mechanisms of abnormal modifications and the safety and effectiveness of targeting them to provide more effective approaches for the prevention and treatment of neurodegenerative diseases.

## Data Availability

Not applicable.

## Abbreviations

PPTMs: Protein post-translational modifications
PTMs: Post-translational modifications
PTM: Post-translational modification
NDDs: Neurodegenerative disorders
AD: Alzheimer’s Disease
PD: Parkinson’s Disease
HD: Huntington’s disease
ALS: Amyotrophic Lateral Sclerosis
Aβ: β-amyloid
GFAP: Glial Fibrillary Acidic Protein
H2S: Hydrogen sulfide
iPSCs: Induced pluripotent stem cells
mTOR: Mammalian target of rapamycin
Ka: Kaempferol
cryo-EM: Cryo-electron microscopy
CRMP2: Collapsin Response Mediator Protein 2
PLG: Piperlongumine
PHB2: Prohibitin 2
LC3: Light chain 3
HATs: Histone acetyltransferases
HDAC(s): Histone deacetylase(s)
DOPAL: 3,4-dihydroxyphenylacetaldehyde
aSyn: α-synuclein
HNE: 4-hydroxy-2-nonenal
HTT: Huntingtin protein
SNP: Single nucleotide polymorphism
polyQ: Polyglutamine
CDK5: Cyclin-Dependent Kinase 5
HDAC6: Histone Deacetylase 6
BDNF: Brain-derived neurotrophic factor
DR: Dietary restriction
UBF-1: Upstream binding factor-1
rDNA: Ribosomal DNA
CBP: CREB binding protein
SIP: Siah-1-interacting protein
AR: Androgen receptor
SBMA: Spinal and bulbar muscular atrophy
SCA3: Spinocerebellar ataxia type 3
Herp: Homocysteine-induced endoplasmic reticulum protein
NLK: Nemo-like kinase
mHTT: Mutant HTT
TMP: Tetramethylpyrazine
PF11: Pseudoginsenoside-F11
DLD: Dihydrolipoamide dehydrogenase
Cin: Cinnamaldehyde
TG: Tripterygium glycoside
TK: Tyrosine kinase
APP: Amyloid precursor protein
hUCB-MSCs: Human umbilical cord blood-derived mesenchymal stem cells
N6FFA: N6-Furfuryladenine
LRRK2: Leucine-rich repeat kinase 2
PTE: Plastrum testudinis extract
SAM: S-adenosylmethionine
COMT: Catechol-O-methyltransferase
PP2A: Protein phosphatase 2 A
NNMT: Nicotinamide N‑Methyltransferase
SOD1: Superoxide dismutase
TSA: Trichostatin A
TFEB: Transcription factor EB
pα-SYN (S129): Phosphorylated α-synuclein at serine 129
XXD: Xixin decoction
SAD: Sporadic Alzheimer disease
